# Supplementary material for: Effect of major ozone autohemotherapy in fibromyalgia syndrome: a retrospective study
Source: PeerJ. 2025 Dec 5;13:e20475. doi: 10.7717/peerj.20475 (PMC12684411; doi:10.7717/peerj.20475)
Supplement: Supplemental Information 2 [file peerj-13-20475-s002.docx]

Codebook

- VASbeforeTreatment : Visual Analog Scala Score Before Treatment
- VASAfterTreatment: Visual Analog Scala Score After Treatment
- fiqbeforeTreatment: Fibromyalgia Impact Questionnaire (FIQ) Score Before Treatment
- fiqAfterTreatment: Fibromyalgia Impact Questionnaire (FIQ) Score After Treatment
- SleepbeforeTreatment: sleep Score Before Treatment
- SleepafterTreatment: sleep Score After Treatment
- sf36physicalfunctioningbeforetreatment: sf36 physical functioning score before treatment
- sf36physicalfunctioningaftertreatment: sf36 physical functioning score after treatment
- sf36rolephysicalbeofretreatment: sf36 physical score before treatment
- sf36rolephysicalaftertreatment: sf36 physical score after treatment
- sf36energyfatigubeforetreatment: sf36 energy/fatigue score before treatment
- sf36energyfatiguaftertreatment: sf36 energy/fatigue score after treatment
- sf36mentalhealthbeforetreatment: sf36 mental health score before treatment
- sf36mentalhealthaftertreatment: sf36 mental health score after treatment
- sf36socialfunctioningbeforeTreatment: sf36 social functioning score before treatment
- sf36socialfunctioningafterTreatment: sf36 social functioning score after treatment
- sf36painbeforeTreatment: sf36 pain score before treatment
- sf36painafterTreatment: sf36 pain score after treatment
- sf36generalhealthbeforetreatment: sf36 general health score before treatment
- sf36generalhealthbeforetreatment: sf36 general health score after treatment
